# Editorial: New Translational Insights on Metabolic Syndrome: Obesity, Hypertension, Diabetes and Beyond

**DOI:** 10.3389/fphys.2016.00229

**Published:** 2016-06-10

**Authors:** Camille M. Balarini, Valdir A. Braga

**Affiliations:** ^1^Health Sciences Center, Federal University of ParaibaJoao Pessoa, Brazil; ^2^Biotechnology Center, Federal University of ParaibaJoao Pessoa, Brazil

**Keywords:** metabolic syndrome, obesity, hypertension, diabetes, atherosclerosis, inflammation

Metabolic syndrome (MetS) can be considered as the clustering of several risk factors such as obesity, hypertension, insulin resistance, and dyslipidemia, which could lead to the development of diabetes and cardiovascular diseases (CVD). The criteria for clinical diagnosis of MetS consist of 3 or more of the following: (1) waist circumference >102 cm in men and 88 cm in women; (2) triglycerides >150 mg/dL; (3) HDL < 40 mg/dL in men and < 50 mg/dL in women; (4) blood pressure ≥ 130/85 mmHg; (5) fasting glucose ≥ 100 mg/dL. Considering that the underlying mechanisms leading to the concurrence of these factors are not yet well-established, the aim of this research topic was to provide a space where researchers holding different backgrounds could shed some light onto the pathophysiology of different risk factors involved in MetS.

The present research topic involves eleven articles including both reviews and original manuscripts. In a translational perspective, the topic includes both clinical and experimental approaches from different research groups located in several countries, which discuss different aspects of MetS such as hypertension, obesity, diabetes, atherosclerosis, and inflammation. At the present moment, almost 3000 article downloads were performed from researchers all over the world. In this editorial, we highlight important insights from these articles leading to a better comprehension of MetS and its complications.

Interestingly, in one aspect of metabolic disorders, the review articles by Costa-Silva et al. and Silva et al. discuss the effects of malnutrition on the establishment of hypertension. Using an *in utero* perspective, Costa-Silva et al. evaluate the effects of maternal malnutrition during pregnancy on sympathetic-respiratory dysfunction that leads to hypertension. It has been shown that maternal low-protein diet during gestation negatively affects organ growth and increases sympathetic tone in the offspring. In addition, Silva et al. focus on post-weaning protein malnutrition and its cardiovascular implications. Authors demonstrated that post-weaning low protein intake impairs cardiovascular reflexes, increases sympathetic tonus, decreases vagal tonus, and enhances arterial blood pressure and heart rate. Both groups agree that increased hypoxia-inducible factor expression in malnutrition is responsible for the enhanced sympathoexcitation arising from chemoreflex activation.

Opposite to malnutrition, obesity was discussed in three independent articles. As highlighted by Del Rio et al., most efforts to understand MetS have been focused on the study of peripheral organ malfunction, while the role of the nervous system on alterations observed in MetS remains poorly understood. Focusing on central mechanisms involved in MetS, Moreira et al., Cruz et al., and Del Rio et al., independently discussed the impact of increased sympathetic activity in obesity. Moreira et al. provided us with a substantial review of important MetS components such as obesity, hypertension, insulin resistance, dyslipidemia, and inflammation. The link between obesity and cardiovascular damage may reside on leptin, since this peptide can increase sympathetic renal activity, reactive oxygen species (ROS) in kidney and reduce nitric oxide and that hyperleptinemia is a common feature in obesity. Regarding central nervous system alterations in MetS, Cruz et al. documented that the sympathoexcitation caused by peripheral Ang II-induced ROS formation along the subfornical organ (SFO) and paraventricular nucleus of the hypothalamus (PVN) may be a putative mechanism to explain the metabolic disorders underlying MetS. Lastly, Del Rio et al. bring a different approach and focus their work on glial cells within central nuclei involved in sympathetic control and how these cells could contribute to the pathogenesis of MetS.

Metabolic syndrome is also involved in impairment of glycemic control and end-organ damage. Therefore, Peleli et al. studied the effects of inorganic nitrate on glucose and insulin signaling in an experimental model of MetS (adenosine receptor type A_2B_ knockout mice, ${\text{A}}_{2\text{B}}^{-∕-}$). Acute nitrate administration to ${\text{A}}_{2\text{B}}^{-∕-}$ mice resulted in ameliorated glucose tolerance, reduced HOMA-IR and increased plasma nitrate. Authors suggested that impaired AMP-activated kinase (AMPK) activation and increased NADPH oxidase in liver could contribute to MetS in this model, since the ratio of phosphorylated/non-phosphorylated AMPK was reduced and NADPH oxidase activity was increased in liver of ${\text{A}}_{2\text{B}}^{-∕-}$ mice.

Considering that diabetic nephropathy is an important cause of death among diabetic patients, Gomes et al. proposed that a low-dose of the antioxidant quercetin could improve metabolic parameters and renal function in an experimental model of diabetes and atherosclerosis (apolipoprotein E knockout—apoE^−∕−^—diabetic mouse). Furthermore, Arsenijevic et al. propose that a primary reduction in kidney function can be considered the cause of a pro-inflammatory and pro-oxidative status, leading to metabolic disorders.

Lastly, three independent research groups focused their work on the implications of inflammation in different determinants of MetS. Firstly, Freitas Lima et al. discussed the influence of adipokines in insulin resistance and atherosclerosis focusing on adiponectin, TNF-α, IL-6, MCP-1, and leptin. Considering that endothelial dysfunction is a *sine qua non* condition to atherosclerosis development, the comprehension of this phenomenon can create a window of opportunities for preventive measures. Is this regard, Kraemer-Aguiar et al. discussed the hypothesis that, as obesity is a multiple grade disease, an increasing impairment of vascular function would occur from lean to severe obese subjects. Using a different perspective, Cavalcante-Silva et al. discussed the influence of gut microbiota in inflammation and MetS. Authors provide us with a broad review on normal gut microbiota and how it can modulate adiposity, glucose homeostasis, fat accumulation, and other metabolic pathways.

In conclusion, we found that this research topic, devoted to a better understanding of MetS, and its associated comorbidities, was very enlightening. We would to like to thank all authors and reviewers that helped us to provide a quality-based topic.

## Author contributions

CB and VB participated in the design of the manuscript, drafted the manuscript, revised it critically and approved the final version.

## Funding

Conselho Nacional de Desenvolvimento Científico e Tecnológico (CNPq), grant numbers 472133/2013-6 and 304772/2014-3 (VB) and Coordenação de Aperfeiçoamento de Pessoal de Ensino Superior (CAPES) (VB).

### Conflict of interest statement

The authors declare that the research was conducted in the absence of any commercial or financial relationships that could be construed as a potential conflict of interest.

